# Hereditary Transthyretin-Related Amyloidosis Ongoing Observational Study: A Baseline Report of the First 3167 Participants

**DOI:** 10.3390/jcm13206197

**Published:** 2024-10-17

**Authors:** Sabine Rösner, Luba M. Pardo, Aida M. Bertoli-Avella, Volha Skrahina, Pierre Engel, Sabine Schröder, Susan Zielske, Valerie Bonke, Janett Kreth, Gina Westphal, Felix Reder, Snezana Skobalj, Susanne Zielke, Xenia Bogdanovic, Paula Grieger, Jörg Rennecke, Thomas Skripuletz, Monica Patten, Birgit Aßmus, Katrin Hahn, Arndt Rolfs, Peter Bauer

**Affiliations:** 1 CENTOGENE GmbH, 18055 Rostock, Germany; sabine.roesner@centogene.com (S.R.); luba.pardo@centogene.com (L.M.P.); aida.bertoli-avella@centogene.com (A.M.B.-A.); skrahinavolha@gmail.com (V.S.); engel.pierre@gmail.com (P.E.); sabine.schroeder@centogene.com (S.S.); susan.zielske@centogene.com (S.Z.); valerie.bonke@centogene.com (V.B.); gina.westphal@centogene.com (G.W.); felix-reder@gmx.de (F.R.); snezanaskobalj@gmail.com (S.S.); susanne.zielke@mail.de (S.Z.); xenia_bogdanovic@web.de (X.B.); paula.grieger@uksh.de (P.G.); j-rennecke@t-online.de (J.R.); arndt.rolfs@web.de (A.R.); 2 Department of Neurology, Hannover Medical School, 30625 Hannover, Germany; skripuletz.thomas@mh-hannover.de; 3 Amyloidosis Center of Lower Saxony, Hannover Medical School, 30625 Hannover, Germany; 4 Department of Cardiology, University Heart and Vascular Center, 20148 Hamburg, Germany; patten@uke.de; 5 Department of Cardiology and Angiology, University Hospital Giessen and Marburg, 35043 Marburg, Germany; birgit.assmus@innere.med.uni-giessen.de; 6 Department of Neurology, Charitè–Universitätsmedizin, Amyloidosis Center Charité, 10117 Berlin, Germany; katrin.hahn@charite.de; 7 Amyloidosis Center Charité Berlin (ACCB), Charité Universitätsmedizin, 10117 Berlin, Germany; 8 RCV GmbH, Institute for Rare Disease Diagnostics, 10629 Berlin, Germany; 9 Medical Faculty, University Rostock, 18057 Rostock, Germany

**Keywords:** hATTR amyloidosis, Hereditary Transthyretin-Related Amyloidosis, *TTR*

## Abstract

**Background**: Hereditary transthyretin-related amyloidosis is a clinically heterogeneous autosomal dominant disease caused by pathogenic variants in the *TTR* gene (hATTR amyloidosis). **Objective:** The current study describes the demographic, clinical, and genetic characteristics of patients with suspected hATTR amyloidosis. **Methods**: This study is part of the “Hereditary transthyretin-related amyloidosis and longitudinal monitoring of *TTR*-positive patients” (TRAMmoni*TTR*) study. This study included 3167 participants, along with their clinical details. Principal component (PC) analysis was used to analyze their clinical symptomatology. Next-generation sequencing of the *TTR* gene was performed and genotype–phenotype relationships were investigated. We compared the demographic and clinical characteristics using the principal components (PCs) and also compared participants with and without the *TTR* pathogenic variants. **Results:** We identified five main clinical phenotypes out of 22 single symptoms that explained 49% of the variation. The first two PCs referred to polyneuropathy and cardiomyopathy. We found significant differences between gender and PC-polyneuropathy and PC-cardiomyopathy, with male over-representation in the higher quantiles of PC-polyneuropathy and male under-representation in the lowest quantiles of PC-cardiomyopathy. We identified 92 participants with hATTR (3%), exhibiting 17 unique heterozygous *TTR* variants. The p.Val50Met variant was the most frequent. Furthermore, 503 participants (20%) were identified with ATTR and no relevant *TTR* variants (ATTRwt). We detected significant differences between the ATTRwt and hATTR groups, with male gender predominance in only the ATTRwt group and a positive family history of polyneuropathy and/or cardiomyopathy among the hATTR participants. **Conclusions**: The current clinical and genetic characterization of this cohort serves as a foundation for further longitudinal monitoring and assessment.

## 1. Introduction

Transthyretin amyloidosis (ATTR amyloidosis) is a life-threatening disease caused by the deposition of misfolded transthyretin (TTR) protein amyloid fibrils in various tissues, leading to polyneuropathy and/or cardiomyopathy, among other clinical manifestations [1,2]. ATTR amyloidosis can be classified into two distinct forms: hereditary or variant ATTR amyloidosis (ATTRv amyloidosis) and wild-type ATTR amyloidosis (ATTRwt amyloidosis). ATTRv amyloidosis is an autosomal dominant disease caused by pathogenic variants in the *TTR* gene, which is located on chromosome 18 [3]. Pathogenic variants causing ATTRv amyloidosis were initially identified in 1984 by Saraiva et al., who reported the heterozygous p.Val50Met variant (legacy Val30Met, NM_000371.4:c.148G>A; hereafter p.Val50Met) in a Portuguese family (4). To date, this is the most frequently reported variant in *TTR* [4]. There are over 200 *TTR* variants reported in the Human Genetics Mutation database HGMD; https://www.hgmd.cf.ac.uk/docs/articles.html (accessed on 19 December 2023). In contrast, ATTRwt amyloidosis is ‘acquired’ and results from the accumulation of the ‘wild-type’ TTR protein. This form of the disease is more common in men and occurs in older age (>60 years) [2].

The phenotypic spectrum of ATTRv amyloidosis is highly heterogeneous and encompasses polyneuropathy, carpal tunnel syndrome, autonomic insufficiency, cardiomyopathy, and gastrointestinal features, occasionally accompanied by vitreous opacities or renal insufficiency. As the disease progresses, gastrointestinal features with malabsorption, cachexia, incapacitating neuropathy, severe cardiac disturbances, and marked orthostatic hypotension become predominant [5]. Broadly, two primary phenotypes are recognized: polyneuropathy (PNP), which is characterized by autonomic dysregulation, cardiomyopathy (CM), or a blend of both neurological and cardiac features (1). Notably, adult-onset CM emerges as the prevailing presentation in ATTRwt amyloidosis, with a predilection in men [1].

The accurate diagnosis of ATTR amyloidosis poses remarkable challenges due to the disease’s clinical heterogeneity, low awareness of the disease, and clinical features that overlap with more prevalent conditions [1], particularly in geographical regions with a low prevalence of the causative variants. A better understanding of the disease holds the potential to facilitate earlier detection and enable timely intervention with approved disease-modifying therapies [6].

A comprehensive longitudinal observational study, the THAOS study, was conducted over a 14-year period [7] and provided valuable insights into the primary clinical features of ATTR amyloidosis, encompassing both the TTRv and TTRwt forms, across diverse geographical regions including North America, South America, Asia, Africa, and Europe. Notably, Portugal and the USA (United States of America) contribute the largest patient cohorts, amounting to 3135 individuals. However, the prevalence of pathogenic variants and the associated clinical manifestations in other regions remains poorly explored.

More recently, a multicentric observational initiative, the “Epidemiological Analysis for the Hereditary TransthyRetin-Related Amyloidosis” (TRAM) study, has been established. This study aimed to investigate the prevalence of hereditary ATTR (hATTR) amyloidosis among participants presenting with polyneuropathy (PNP) and/or cardiomyopathy (CM) who lacked an obvious etiology [8]. By broadening the scope of the investigation to encompass more regions and by focusing on participants with specific clinical presentations, the TRAM study seeks to enhance our understanding of hATTR prevalence and its associated clinical spectrum beyond previously studied populations.

A newly launched study, “Hereditary transthyretin-related amyloidosis and longitudinal monitoring of *TTR*-positive patients” (TRAMmoni*TTR*), is a continuation and expansion of the preceding TRAM epidemiological analysis [8]. It is expected to enroll a sizable cohort of 5000 participants from Germany. This ongoing study aims to delve deeper into the understanding of ATTRv amyloidosis and its progression (hereafter, we use hATTR to refer to ATTRv). In this paper, we describe the main demographic and clinical characteristics at baseline and *TTR* genetic results of the first 3167 participants. This characterization of the cohort serves as a foundation for longitudinal monitoring and analysis.

## 2. Materials and Methods

### 2.1. Study Design

The genetic screening of an at-risk population for hereditary transthyretin-related amyloidosis and the longitudinal monitoring of *TTR*-positive subjects (the TRAMmoni*TTR* study) is a German epidemiological, multicentre, cohort study of participants at risk for hATTR, along with participants already diagnosed with hATTR. The list of centers and researchers involved in this study is provided in Table S1. The follow-up of the clinical status covers 24 months (about 2 years). The TRAMmoni*TTR* study was designed as an extension of the earlier TRAM analysis epidemiological study [8] (concluded in September 2020) to obtain a deeper understanding of the disease. The main aim of the TRAMmoni*TTR* study is to monitor the clinical status of subjects who have tested positive for *TTR*, and the secondary aim is to explore hATTR prevalence in an at-risk population. The tertiary objective is to identify hATTR biomarkers. A detailed protocol is accessible from Clinical.Trials.gov; reference: NCT03237494; https://clinicaltrials.gov/search?cond=NCT03237494 (accessed on 19 December 2023).

This ongoing study, started in October 2020 and with an expected conclusion in April 2025, aims to establish a cohort including 5000 participants. For this paper, we have included data derived from two distinct sources. Firstly, participants enrolled in the TRAMmoni*TTR* study between February 2021 and September 2023 form cohort 1 (*n* = 2518), with both clinical data and results from *TTR* genetic testing. Secondly, individuals included in the TRAM analysis, recruited from October 2020 to May 2021, constitute cohort 2 (*n* = 649), for whom both clinical and genetic data were also included. The inclusion criteria for the TRAM study were described in a previous publication [8]. These were as follows: (1) informed consent was obtained from the participant, (2) the participant was aged between 18 and 85 years, (3) the participant was diagnosed with CM and/or PNP of no obvious etiology, and (4) the participant has not undergone chemotherapy for any solid or hematological malignancies. The two cohorts were combined, given the similar clinical inclusion criteria. This current study is an in-depth examination of the clinical and genetic features seen across this cohort of 3167 participants.

### 2.2. Ethical Approval

The research was carried out in accordance with the Declaration of Helsinki. The TRAMmoni*TTR* study was approved by the Ethical Committee of the Medical Faculty of the University of Rostock (approval A 2018-0069, date: 24 April 2018). Ethics approval was obtained for each participating center in Germany [6]. Informed consent was obtained from the referring physicians, patients, and/or their parents or legal guardians, including regarding the publication of anonymized individual details. The informed consent form is available in English and in several other languages at: https://www.centogene.com/downloads.html (accessed on 19 December 2023).

### 2.3. Description of the Clinical Cases

Inclusion criteria to participate in cohort 1 were: (i) age of 18 years and older; (ii) the participant had provided signed, informed consent; (iii) the participant had not undergone chemotherapy treatment for any carcinoma; (iv) the participant had not had any diagnosis of alcoholism according to international guidelines; and (v) the participant had at least two symptoms of the following: cardiomyopathy or polyneuropathy with no obvious etiology, atypical chronic inflammatory demyelinating polyneuropathy (CIDP) or motor neuron disease (MND), autonomic dysfunction, hypertrophic cardiomyopathy or heart failure with preserved ejection fraction left ventricular hypertrophy (LVH), bilateral carpal tunnel syndrome, spinal stenosis or spinal radiculopathy, gait disorders, ocular changes involving vitreous opacities, unexplained weight loss (>5 kg), renal abnormalities, a family history of hATTR, the suspicion of ATTRwt amyloidosis based on imaging or biopsy, and not genetically tested for hATTR. In addition, either the participant has already received a diagnosis of hATTR, or the participant is a first- or second-degree relative of a patient diagnosed with hATTR in the study.

The eligible participants for this study indicating a risk of hATTR amyloidosis were genetically tested (see below). Those diagnosed with hATTR prior to enrolment underwent confirmatory genetic tests. Symptomatic or asymptomatic first- and second-degree family members of newly diagnosed participants who tested positive for hATTR were tested as well. The inclusion criteria for cohort 2 were the same as those from the previous epidemiological study (TRAM analysis) [8].

### 2.4. Genetic Testing

All subjects gave blood samples (~1 mL blood), drawn into a dried blood spot (DBS) filter card known as a CentoCard^®^ (CENTOGENE GmbH; Rostock, Germany). These cards were then sent to CENTOGENE laboratories (Rostock, Germany). Concurrently, the participants’ past medical history, medication, family history, and physical examination findings were recorded as an electronic case report form (eCRF). Upon arrival at CENTOGENE’s laboratory, a sequencing analysis of the *TTR* gene (NM_000371.3) was performed. DNA was extracted from the CentoCard^®^ using the Extracta DNA Prep Kit (QuantaBio, Beverly, MA, USA). The DNA was used in a multiplex PCR, generating 12 TTR-specific amplicons covering all coding exons and at least 20 bp exon-intron boundaries. Amplicons were pooled together and purified using AMPure XP beads (Beckman Coulter; Woerden, the Netherlands). A second PCR amplification procedure was performed, adding Illumina-compatible, patient-specific barcoded index primers to each amplicon pool. Following a second bead purification process, the samples were pooled for 150 bp paired-end sequencing on a MiSeq or NextSeq 500 instrument (Illumina Inc; München, Germany). The reads were aligned with bioinformatics methods and compared to the reference sequence *TTR* (NM_000371.3) to identify the single-nucleotide variants (SNVs). All variants with a minor allele frequency (MAF) of less than 1% in the gnomAD database, and disease-causing variants reported in HGMD^®^; https://www.hgmd.cf.ac.uk/ac/index.php (accessed on 19 December 2023) in ClinVar, or in the CENTOGENE Biodatabank were evaluated. All variants were classified according to the general recommendations of the American College of Medical Genetics ACMG/AMP ClinGen Sequence Variant Interpretation.

The *TTR* test results were interpreted as positive (pathogenic or likely pathogenic—P/LP—variant), as unclear (variant of unknown significance—VUS), or as negative when no relevant variant was identified. Participants with an established/possible genetic diagnosis (positive *TTR* result or unclear result) were then further engaged in the study. First- and second-degree relatives of participants with a P/LP or VUS in the *TTR* gene were invited to the study site by a doctor connected to the study. The clinical history of subjects was thoroughly examined, and symptomatic and asymptomatic family members were offered enrollment in the TRAMmoni*TTR* study with subsequent genetic testing. Asymptomatic family members were given the option of genetic counseling before predictive genetic testing for hATTR. If testing was chosen, blood was collected and sent to CENTOGENE, with all participating family members receiving new study codes and their relation to the index subsequently documented.

*TTR*-positive subjects, both symptomatic and asymptomatic, or with VUS in their *TTR* gene, were offered enrollment in a 24-month follow-up program. Over eight visits, each encompassing a physical examination, clinical information documentation, and blood sample collection, participants underwent biochemical analysis to establish their hATTR biomarkers.

## 3. Statistical Analysis

Descriptive statistics of the main demographic variables were presented as medians with inter-quantile range (IQR) and compared using the chi-squared test for categorical variables and the Mann–Whitney test for continuous variables. Cross tables were used to analyze the categorical data and differences across variables were tested using the chi-squared test, adjusting for post hoc pairwise analysis (correcting for multiple testing). All tests were considered significant if the *p*-values (or adjusted *p*-values) were lower than or equal to 0.05.

To understand the variations in the clinical symptoms of the participants of both cohorts, we used principal component analysis (PCA) as an exploratory analysis method (the results of the PCA are presented in the Supplementary Materials). This is a multivariate reduction technique used to identify common variations across correlated variables to select the main components (latent variables) that explain most of the variation across the original data. The principal components (PCs) are new variables built as a combination of the original variables. The PCs are built in such a way that the first PC explains most of the variation of the data, the second PC is uncorrelated with the first one and explains the variations not accounted for by the first PC, and so on. The aim is to identify which components explain most of the variations of all data with the minimum number of PCs. Those PCs that explain most of the variation are retained for further analysis, while those that do not explain a lot of the variation are discarded. To decide on which PCs were relevant, we used the Kayser criterion and selected PCs with an eigenvalue of ≥1 [9]. Furthermore, we used the score distribution of each PC to categorize them into quantiles (Figure S1). The aim was to assign the participants to the highest quantiles (Q3–Q4) or lower quantiles (Q1–Q2). Individuals in the highest quantile will have more of (or a combination of) the relevant symptoms than those in the lower quantiles. This implies that individuals occupying the upper quantiles are more likely to have a combination of clinical symptoms that are indicative of the severe end of the clinical spectrum.

For this analysis, we defined the clinical symptoms as ‘0’ and ‘1’ to denote the absence or presence of symptoms, respectively. PCA was carried out using matrix correlations on complete cases and using varimax rotation, wherein the residual correlation between latent components is not assumed. The above analysis was performed using SPSS for Windows, version 21.0 (SPSS; Chicago, IL, USA). Furthermore, we compared the quantile distribution of the PCs (Q1; Q2; Q3; Q4) according to their demographic and genetic characteristics. Overall differences in the observed versus the expected distribution (counts of participants per quantile) of the participants for each PC across quantiles per category (e.g., gender; family history of PNP) were determined using chi-square tests. Post hoc analysis was performed when the chi-square value was significant (*p*-value ≤ 0.05) to analyze which group was leading the deviation in the observed–expected distribution of counts. Significant deviations were considered significant with an adjusted *p*-value of ≤0.05.

For the genetic analysis, we considered individuals to be either *TTR*-positive or negative (see Section 2.4, Genetic Testing). We also compared *TTR*-specific P/LP variants with the new PC components. Subjects with VUS were not included in the analysis, due to the uncertain functional and clinical impacts of the variants and the low number that was detected (only 4 participants).

Sensitivity analysis was performed to compare the main demographic and clinical characteristics (using the main PCs). Participants with confirmed ATTRwt amyloidosis (inclusion criteria: ‘suspicion of ATTRwt amyloidosis, based on imaging or biopsy’ and being genetically TTR-negative, *n* = 503) and participants with the clinical suspicion of hATTR and who were TTR-positive (*n* = 92) were included in this analysis.

## 4. Results

### 4.1. Description of the Cohorts

Table 1 presents the characteristics of the dataset and the two cohorts. Cohort 1 consisted of 2518 individuals and cohort 2 consisted of 649 participants. The participants of cohort 2 were slightly younger, with a median age of 64 years (inter-quantile range: 53–75 years old) compared to cohort 1, with a median age of 68 years (inter-quantile range: 56–79 years). In both cohorts, there were more males than females (66% and 64% males in cohort 1 and cohort 2, respectively), but there were no significant differences in the proportion of females between cohorts. Most of the participants were of European ancestry (97% and 94%) with a significantly higher proportion of these in Cohort 2. More than half of the participants were overweight or obese, with an increased proportion of them in Cohort 2. There was a significant difference in the proportion of men in the overweight group, with an under-representation in the underweight and normal weight groups (Supplementary Figure S2; chi-square test: 111.01, df = 3, *p*-value < 2.2 × 10^−16^).

Approximately 50% of the participants could give a recorded age for onset and an age at diagnosis of either CM or PNP. The age of onset and diagnoses of PNP were similar in both cohorts, with a median age of 59 years (IQR: 50–59 years). Participants from cohort 1 had a significantly later age of onset and diagnosis of CM (onset at 71 years (58–79) vs. 65 (55–75)) (Table 1). In both cohorts, the time that elapsed between the onset and diagnosis of CM or PNP was approximately 2 years. The proportion of participants with a familial history of CM or PNP was low, with a similar proportion in both cohorts (9% and 10%, Table 1).

In cohort 1, the largest group of participants had a clinical history of PNP (1477 out of 2537; 58% of participants), while 1038 (of 2537; 41%) had CM and 278 (11%) had both. Such information regarding cohort 2 was unavailable.

### 4.2. Clinical Manifestations

**Figure 1:**
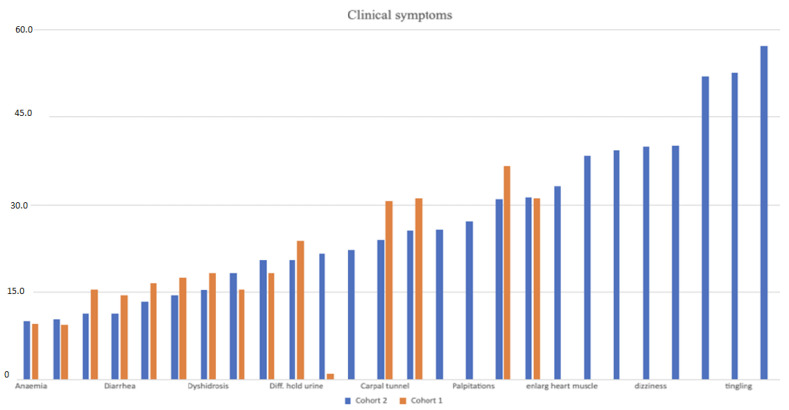
Frequency bar-plot of the main symptoms of participants from the two cohorts described in TRAMmoni*TTR* study 1.

Following the previous study by Skrahina et al. [8], we estimated the prevalence of symptoms associated with PNP and CM (Figure 1). In cohort 1, the most common symptoms included tingling and numbness (67%), fatigue (47%), and weakness (45%), and less common symptoms included a loss of smell (6%) and enlargement of the heart (1%). In cohort 2 the main symptoms were: pain, tingling, and numbness present in over 50% of participants. Less common symptoms included anemia (10%), bouts of constipation and diarrhea (10%), and erection problems (75% of men).

Cohort 1 consists of 2518 participants from the TRAMmoniTTR study and cohort 2 consists of 649 participants from the TRAM study.

To understand the underlying physiopathology of the clinical symptoms in participants from the TRAMmoni*TTR* study, we performed a PCA using the shared symptoms in both cohorts. Out of the shared 22 individual symptoms, we excluded the HPO term ‘enlarged heart muscle’ since it was reported in few participants from cohort 1. In addition, we excluded the HPO term ‘difficulty in getting an erection’, since this was only restricted to men.

Details of the PCA analysis are presented in the Supplementary Results (Table S2: Descriptive statistics of main symptoms used for PCA), Table S3 (KMO and Bartlett’s test, used to assess whether PCA was appropriate for the dataset). In the factor analysis, we identified five main variables (hereafter principal components—PCs; eigenvalue > 1) that explained up to 49% of the common variation in the symptoms in the 2345 cases with complete data (Table 2; Table S4). Looking at the correlation coefficients between individual symptoms and the identified clusters showed us that there was a high correlation between the first PC and polyneuropathy-associated symptoms, including the dysregulation of temperature (0.75), the sensation of burning feet (0.73), allodynia (0.66), numbness/tingling (0.66), and dyshidrosis (0.44). We labeled this component as polyneuropathy-related (PC-NP, Table 2). The second PC was dominated by cardiovascular symptoms, including palpitations (0.81), heartbeat problems (0.71), and shortness of breath (0.62), with moderate correlations with chest pain (0.52), and a weak correlation was detected with water retention in the ankles and limbs (0.36). We labeled this component as cardiomyopathy-related (PC-CM; Table 2). The third variable was labeled as a gastrointestinal component (PC-GI; Table 2) with high correlations for constipation (0.65), diarrhea (0.74), or bouts of diarrhea or constipation (0.82), and a weak correlation with difficulty in holding back urine (0.36). General complaints (PC-GC) were correlated with unintended weight loss (0.75) and anemia (0.68). Finally, carpal tunnel syndrome (PC-CT) was mostly dominated by this pathology (0.79) and water retention in the ankles and limbs (0.53).

We used the quantile distribution of the PCs to analyze the data. Figure S1 presents the score distribution for four main PCs, alongside the participants per quantile. This comparison allowed the evaluation of the observed number of individuals per quantile (Section 2, Materials and Methods) with other demographic characteristics and focused on the four main PCs.

We found significant differences between gender and PC-NP, PC-CM, and PC-GI, with an over-representation of men in the higher quantiles of PC-NP (relative to the lower quantiles; Table S6; chi-squared *p*-value = 5 × 10^−12^), while the opposite result was found for women. This means that more men were in the more severe clinical spectrum of PNP while women tended to be at the milder end of the spectrum.

In addition, there was an under-representation of men in the lowest quantiles for PC-CM (chi-squared *p*-value = 4.893 × 10^−12^).

We also found significant differences when comparing participants with PNP regarding their family history of PNP. Overall, we found that among the participants in the group with a positive family history, there was a higher number (than expected) of participants at the mild end of the clinical spectrum, with a trend toward a lower number of participants at the more severe end of the spectrum (Table S6). Similarly, we detected significant differences when comparing participants with CM and their family history of this disorder. Among patients with a positive family history of CM, there was an over-representation of participants at the milder end of the CM clinical spectrum (Table S6). This could suggest that having a positive family history leads to earlier disease recognition.

### 4.3. Genetic Analysis

After sequencing analysis of the *TTR* gene in all participants, we detected that 3% of the participants (*n* = 92/3206) were positive for heterozygous P/LP variants in *TTR*. We identified 17 different missense variants. Of these, the most common variant was c.148G>A p.Val50Met, which was present in 42% of the participants who were positive for *TTR* variants. The second most frequent variant was c.424G>A p.Val142Ile (hereafter: p.Val142Ile), which was present in 13%, followed by c.311T>C p.Ile104Thr (hereafter: p.Ile104Thr), which was present in 9% of the *TTR*-positive participants. The remaining variants were found in fewer than nine participants (Table 3). There were only four VUS reported: c.280G>C p.Asp94His [10]; c.386C>T p.Ala129Val [11]; c.310A>G p.Ile104Val; and c.427A>G p.Thr143Ala. The last two variants are novel and ultra-rare and are reported for the first time in this study, with no occurrences in gnomAD.

We tested for differences in the number of participants at different quantiles of the main PCs and TTR-positive (hATTR = 92) and TTR-negative participants (ATTRwt = 503). We found differences in *TTR* status and the PC-GI (chi-squared test: 9.3; 3 df; *p*-value = 0.026), with a tendency to a lower proportion of *TTR*-positive individuals in the highest quantile (Table S6). Thus, participants with hATTR were less likely to have severe GI symptoms. We also found borderline differences per gender and BMI categories in hATTR vs. ATTRwt participants (chi-square test: 3.68; 1 df; *p*-value = 0.06 for gender), with a higher proportion of males in the hATTR group, although this was not significant after correcting for multiple testing. For the BMI groups, we found a borderline significance difference in *TTR*-positive status (chi-squared: 7.4783, df = 3, *p*-value = 0.06 for BMI categories), with an over-representation of hATTR in the overweight group, although this was not statistically significant in the post hoc analysis.

**Figure 2:**
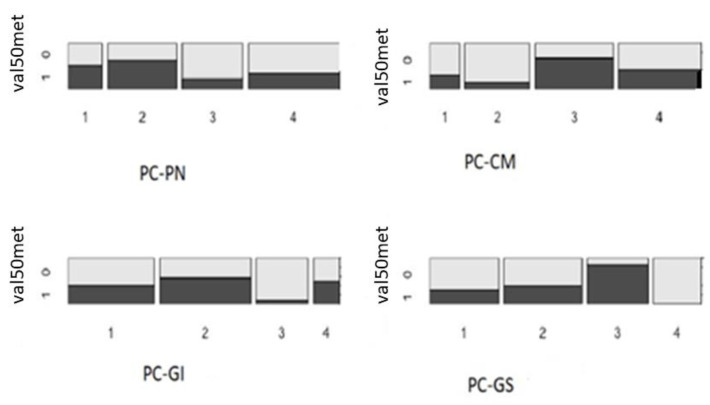
Distribution of TTR p.Val50Met heterozygotes and TTR+ without the p.Val50Met variant per quantile (1–4) of the main principal components (PC).

Furthermore, we selected only *TTR*-positive subjects (hATTR) and compared whether there were differences in the demographics or clinical characteristics of subjects with specific P/LP variants. Given the low prevalence of *TTR* P/LP variants, we focused on the most frequent variant (p.Val50Met; heterozygotes labeled as 1, *n* = 39; no-Val50Met, labeled as 0, *n* = 49). Looking at the main PC variables, there was a significant difference in the number of individuals positive and negative for this variant (chi-squared test = 10.1, df = 3, and *p*-value = 0.02) (Figure 2), with a borderline-significant over-representation of p.Val50Met heterozygotes in the highest quantiles of the PC-CM (Q3-CM, *p*-value = 0.06). Looking at the specific symptoms represented in PC-CM showed an excess of p.Val50Met heterozygotes in participants who reported heartbeat alterations (Fisher’s exact test; *p*-value = 0.05; data not shown).

The graph presents the proportion of participants that were heterozygous for the p.Val50Met variants (labeled as 1) versus TTR+ participants without the p.Val50Met variant (labeled as 0), according to the main principal component of the study, namely, the polyneuropathy (PN), cardiomyopathy (CM), gastrointestinal (GI), and general symptoms (GS) components.

Finally, we compared the demographic and clinical characteristics of participants with hATTR (*TTR*-positive) and participants with ATTRwt (*TTR*-negative and also meeting the inclusion criteria for ‘suspicion of amyloidosis, based on imaging or biopsy’). We found a significantly increased number of men in the ATTRwt group compared with the hATTR group (Table S7). From these analyses, we concluded that there was an over-representation of hATTR patients in the lower clinical spectrum of PNP, with an opposite effect seen in the ATTRwt group. In addition, there were fewer than expected hATTR patients in the lower clinical spectrum of CM, compared to the ATTRwt group, which had more than the expected number of patients in the lower CM clinical spectrum.

Furthermore, there was a significant over-representation of hATTR participants with a positive family history of CM (chi-square = 64.8; df = 3; *p*-value = 8.024 × 10^−16^) and PNP (81.60; *p*-value < 2.2 × 10^−16^) (Supplementary Table S6), as expected, given the autosomal dominant inheritance of the disorder.

## 5. Discussion

For this study, we described the main demographic and clinical characteristics of 3167 study participants who were at risk of hATTR amyloidosis, as part of the TRAMmoni*TTR* study, which included *TTR* genetic testing of this at-risk population. Over half of the participants of this study were men who were overweight or obese (Supplementary Figure S1), who presented with symptoms consistent with PNP (59%). Genetic analysis of *TTR* showed that 3% of the participants had P/LP variants, with p.Val50Met, p.Val142Ile, and p.Ile104Thr being the most prevalent (42%, 11%, and 9%, respectively). We also identified two novel variants (of unknown significance—VUS); c.310A>G p.Ile104Val and c.427A>G p.Thr143Ala have not been described previously in the literature or in public databases.

The demographic characteristics of the present study (*n* = 3167 participants from cohort 1 and cohort 2) were in line with the previous TRAM study, with the disease being more prevalent in men than in women (66% males in the TRAMmoni*TTR* study vs. 62% of males in the TRAM study). The median age of the participants in the current study was 67 years (IQR: 55–78, TRAMmoni*TTR* study) vs. 61 years (IQR: 18–85) in the TRAM study. The demographic characteristics in this work were also similar to the THAOS study, although the proportion of men in the THAOS study was much larger (70%) and included a wider range of participants from different countries [7].

Clinically speaking, from the total of 2537 participants with a clinical history of PNP and/or CM, 58% presented with PNP, 41% presented with CM, and 11% presented with both. Previously reported studies showed a variable frequency of PNP (THAOS 64%, TRAM 23.8%) [8,12]. CM was reported at low frequency among THAOS subjects (14.9%) compared to TRAM (55.4%), while the frequency of combined PNP and CM was similar in both studies (21.1% and 20.8%) [8,12]. Differences in the frequency of PNP, CM, and combined phenotypes are likely due to the participants’ geographical origin and the inclusion criteria. The THAOS report included 1411 symptomatic subjects from nine continental Western European countries [12], with a large proportion of patients from Portugal, where the disease is considered to be endemic. In addition, it has been suggested that the decreased proportion of CM patients in the THAOS study could be due to bias in the diagnostic assessment process, which included more neurology specialists in Portugal [13]. In contrast, the TRAM study included 5141 participants who enrolled in Germany, Austria, and Switzerland [8], with the involvement of both neurological and cardiovascular specialists across the recruitment centers.

Historically, ATTR amyloidosis has been classified as ATTR-PNP, ATTR-CM, or a mixture of both phenotypes, depending on the clinical features and the results from diagnostic tests. In the current study, we applied PCA to cluster single symptoms into main groups to enable genotype–phenotype analyses. In this data-driven analysis, these two main phenotypes were also retrieved. Moreover, we identified a third component of mostly gastrointestinal symptoms. This component encompassed the clinical symptoms of diarrhea and/or constipation (Table 2). Since PCA is data-specific, it is not clear whether these other components will be relevant in other clinical cohorts. However, the fact that we retrieved the first two main components serves as a first validation for these analyses.

The prevalence of the *TTR* P/LP variants was higher in our study (3%), most likely due to the current inclusion criteria (one criterion was being diagnosed with hATTR amyloidosis—see Section 2). Similar to other studies, the most frequent pathogenic variant was p.Val50Met, with a prevalence of 42%, which is higher than in previously reported studies (pooled prevalence 33%; confidence interval: 16–56; Delgado D et al., November 2023, 4th International Congress of ATTR Amyloidosis). This variant has been associated with an increased prevalence of PNP and the earlier onset of disease [2]. Here, we did not find any significant difference in the number of heterozygotes with p.Val50Met and PNP. Only a borderline difference was observed in male participants with CM (PC-CM), specifically, with alterations in heartbeat. The second most common variant was the p.Val142Ile, with a frequency of 13%, which was the second most frequent variant in the TRAM study (15%). In this study, the p.Ile104Thr was the third most frequent variant, with a frequency of almost 10% (9 participants). This variant has been reported in both German and British patients [14] and has been associated with CM [14]. Due to the low number of participants with this variant, it was not possible to correlate this with other demographic characteristics. In the previous TRAM analysis, this variant had a lower prevalence of 4% (2/55 *TTR*-positive patients). The difference in the prevalence of these variants may be related to regional differences.

We found borderline correlations between sex and presenting more symptoms of PNP with an increased proportion of men in the highest quantile of PNP (PC-NP), and with women having fewer CM and GI symptoms than men (Table 3). We also found that participants with hATTR were less likely to have severe GI symptoms (Supplementary Table S6). This contrasts with previous reports wherein a higher prevalence of GI symptoms in patients with hATTR was reported [15,16]. The clinical presentation of GI may differ in each study, making the comparisons challenging. In general, the prevalence of GI symptoms may be lower, as shown by a recent literature review [17]. Further studies harmonizing the prevalence of GI symptoms may be required to validate our observations.

Finally, participants with a positive familial history of PNP or CM were significantly more likely to have PNP, CM, and GI symptoms themselves. The prevalence of hATTR (3%) in the TRAMmoni*TTR* study showed that most participants either had ATTRwt amyloidosis (20%; 503/2518 from cohort 1) or other types of PNP or CM. Having a positive family history of CM or PNP is a strong indicator of hATTR, and genetic TTR testing should be systematically offered to this group of patients.

The analysis of the hATTR and ATTRwt participants in this study showed a higher proportion of men in the latter group. Importantly, the hATTR group had a similar number of men/women. Regarding their clinical presentation, there was an excess of hATTR patients with few PNP symptoms, with an opposite effect seen in the ATTRwt group. This could imply that at least a group of participants with the inherited or genetic form of ATTR are detected earlier. Diagnosing ATTR amyloidosis presents challenges, as cardiac symptoms may mimic those of more common heart conditions. Traditionally, diagnosis required an invasive and costly endomyocardial biopsy, although this requirement has changed recently with the introduction of non-invasive techniques including scintigraphy in 2010 [18], as well as other echocardiographic techniques used to measure cardiac function. Indeed, in recent publications, it has been shown that measuring both atrial and ventricular cardiac work can help distinguish between the different types of hypertrophic cardiomyopathies [19,20]. In addition, cardiac magnetic resonance has been used to assess the degree and distribution of cardiac hypertrophy and could also help in the differential diagnosis of hypertrophic cardiomyopathy [21].

Still, the broad range of symptoms observed in the participants at the time of enrollment highlights the necessity of a multidisciplinary approach to managing all types of ATTR amyloidosis [2]. Our results seem to indicate a short time window of 2 years between the onset of symptoms and diagnosis in Germany, compared to the THAOS registry, where a 4-year time window was observed [7]. According to this study, the average time to diagnosis after symptom onset was 2 years. The diagnosis of ATTR amyloidosis is frequently delayed by several years. However, as shown in this study, genetic testing in participants with suspected ATTR amyloidosis and a positive family history of PNP or CM will help to distinguish those with the hereditary form of the disease.

One of the study’s strengths is the large sample size and the homogeneous ethnicity of the participants, which allows for the assessment of clinical symptoms and the presence of the relevant *TTR* variants in participants with suspected amyloidosis in an unbiased way. The main phenotypes identified were based purely on statistical analysis and, thus, allowed us to identify other components not currently being considered in other studies. Despite the large sample size, our study is still underpowered for conducting a more comprehensive analysis among hATTR participants and their clinical phenotypes. A meta-analysis of clinical cohorts will be necessary to further assess the phenotype– genotype correlations. Another possible limitation is that the inclusion criteria did not consider deeper phenotyping on neuropathy-related signs such as tendon tenosynovial involvement or lumbar stenosis. Likewise, classifications for cardiac hypertrophy according to the New York Heart Association were not available. Still, with our study, we were able to capture the main features associated with ATTR amyloidosis.

## 6. Conclusions

In this study, we detected a prevalence of 3% of hATTR and 20% of ATTRwt. *TTR* genetic testing should be offered to patients with clinical suspicion of amyloidosis and a family history of CM or PNP. The timely indication provided by genetic testing helped to shorten the diagnostic odyssey in this patient-screening program to roughly two years from symptom onset. This is a relevant achievement since specific treatments are available for both hATTR and ATTRwt [22]. Larger studies will need to be conducted to understand *TTR* genotype–phenotype correlations. The current clinical and genetic characterization of the cohort serves as a foundation for further longitudinal monitoring and assessment.

## Figures and Tables

**Table 1:** Demographic characteristics of the participants from the TRAMmoniTTR study.

Characteristics	Cohort 1 (*n* = 2518)	Cohort 2 (*n* = 649)	*p*-Value Cohort 1 vs. Cohort 2	Total (*n* = 3167)
**Median age in years (IQ)**	68 (56–79)	64 (53–75)	0.001	67 (55–78)
**Missing**	35	0		35
**SEX**			0.07	
**Males**	1663 (65.5%)	424 (63.7%)		2087 (65.9%)
**Females**	853 (33.6%)	224 (33.6%)		1077 (34%)
**Missing**	2 (0.8%)	1 (0.2%)		3 (0.1%)
**ETHNICITY**			0.006	
European ancestry *	2470 (97.4%)	626 (94%)		3095 (97.7%)
**Other**	48 (1.9%)	22 (6%)		69 (2.2)
**Median BMI**	26.0 (23.5–29.2)	26.3 (23.7–30.2)	0.07	26.03 (23.5–29.4)
BMI Categories *			0.006	
**Underweight**	60 (2.4%)	8 (1.2%)		68 (2.2%)
**Normal weight**	988 (39.2%)	233 (35.9%)		1221 (38.6%)
**Overweight**	938 (37.3%)	235 (35.2%)		1173 (37%)
**Obese**	508 (20.2%)	167 (25.7%)		675 (21.3%)
**Missing**	24 (1.0%)	6 (0.9%)		30 (0.9%)
Age at onset of polyneuropathy; Median *	N = 1216; 56 (48–68)	N = 390; 55 (45–65)	0.39	N = 1606; 56 (47–67)
**Age at diagnosis of polyneuropathy; Median**	N = 1188; 58 (50–70)	N = 386; 57 (48–68)	0.08	N = 1574; 59 (50–69)
**Age at onset of cardiomyopathy; Median (IQ)**	N = 721; 71 (58–79)	N = 222; 65 (55–75)	0.001	N = 943; 70 (56–78)
**Age at diagnosis of cardiomyopathy; Median, (IQ)**	N = 761; 73 (58–80)	N = 241; 66 (55–77)	0.001	N = 1002; 71 (57–80)
Familial history of polyneuropathy *			0.001	
**Yes**	232 (9.2%)	60 (9.2%)		292 (9.2%)
**No**	1526 (60.6%)	458 (68%)		1984 (62.6%)
**Unknown**	755 (30%)	127 (19%)		888 (27.8%)
**Missing**	5 (0.2%)	4 (0.6%)		9 (0.3%)
Familial history of cardiomyopathy *			0.001	
**Yes**	226 (9.0%)	67 (10.3%)		293 (9.3%)
**No**	1452 (58%)	427 (65.8%)		1879 (59.3%)
**Unknown**	836 (33.2%)	151 (23.3%)		987 (31.2%)
**Missing**	4 (0.2%)	4 (0.6%)		8 (0.3%)

*p*-values were derived with the chi-squared test or Mann–Whitney tests (see Section 2). *p*-values of <0.05 were considered statistically significant.

**Table 2:** Main variables identified after factor analysis. Five main PCs were identified. The values in the cells are the correlation coefficients between individual symptoms and the main PC identified.

	Principal Component (PC)				
	Polyneuropathy (PC-PN)	Cardiomyopathy (PC-CM)	Gastrointestinal (PC-GI)	General Complaints (PC-GP)	Carpal Tunnel Synd. (PC-CT)
**Carpal tunnel**	0.003	0.007	0.043	−0.049	**0.793**
**Water retention: ankle, limbs**	−0.041	0.358	0.038	0.198	0.533
**Shortness of breath**	−0.224	**0.623**	0.022	0.125	0.305
**Difficulty in holding back urine**	0.139	0.059	0.362	0.070	0.262
**Numbness or tingling**	**0.667**	−0.174	0.068	0.003	0.257
**Burning feet**	**0.726**	−0.055	0.074	−0.061	0.110
**Chest pain**	−0.019	0.518	0.146	−0.122	0.087
**Constipation**	0.079	0.056	**0.667**	0.067	0.067
**Anemia**	−0.030	0.128	0.020	**0.678**	0.064
**Heartbeat alterations**	0.014	**0.717**	0.037	0.151	0.031
**Unintended weight loss**	0.020	−0.054	0.123	**0.747**	0.017
**Dizziness**	0.219	0.288	0.238	0.227	0.009
**Allodynia**	**0.630**	0.067	0.047	−0.014	−0.124
**Sensing temperature alterations**	**0.752**	−0.064	0.031	−0.029	−0.083
**Palpitations**	0.028	**0.809**	0.047	0.003	−0.079
**Dyshidrosis**	0.410	0.108	0.178	0.185	−0.073
**Constipation/diarrhea bouts**	0.088	0.083	**0.833**	−0.011	−0.034
**Diarrhea**	0.033	0.080	**0.753**	0.079	−0.020

Robust correlations (>0.6) between individual symptoms and PC are presented in bold.

**Table 3:** Relevant TTR variants identified in the TRAMmoni*TTR* study (NM_000371.3).

HGVS-cDNA	HGVS-Protein	Variant Classification	Allele Count (All Are Heterozygous)	Frequency
**c.148G>A**	p.Val50Met	Pathogenic	39	42.39
**c.424G>A**	p.Val142Ile	Pathogenic	12	13.04
**c.311T>C**	p.Ile104Thr	Pathogenic	9	9.78
**c.238A>G**	p.Thr80Ala	Pathogenic	6	6.52
**c.379A>G**	p.Ile127Val	Pathogenic	5	5.43
**c.424G>C**	p.Val142Leu	Likely pathogenic	5	5.43
**c.88T>C**	p.Cys30Arg	Pathogenic	4	4.35
**c.118G>A**	p.Val40Ile	Pathogenic	2	2.17
**c.341T>C**	p.Val114Ala	Pathogenic	2	2.17
**c.193G>A**	p.Ala65Thr	Likely pathogenic	1	1.09
**c.224T>G**	p.Leu75Arg	Pathogenic	1	1.09
**c.280G>C**	p.Asp94His	Uncertain Significance	1	1.09
**c.310A>G**	p.Ile104Val	Uncertain Significance	1	1.09
**c.325G>A**	p.Glu109Lys	Pathogenic	1	1.09
**c.377C>A**	p.Thr126Asn	Likely Pathogenic	1	1.09
**c.386C>T**	p.Ala129Val	Uncertain Significance	1	1.09
**c.427A>G**	p.Thr143Ala	Uncertain Significance	1	1.09
**Total**			**92**	100

HGVS nomenclature (Human Genome Variation Society).

## Data Availability

Data are available upon reasonable request. All data relevant to the study are included in the article or in the Supplementary Information. The datasets supporting the current study are not public, due to data privacy laws for patients. Some data may be available upon request to the corresponding author.

## Supplementary Materials

The following supporting information can be downloaded at: https://www.mdpi.com/article/10.3390/jcm13206197/s1, Table S1: Centers participating in the study; Table S2: Descriptive statistics of the main symptoms used for PCA; Table S3: Test to assess whether the data are suitable for PCA; Table S4: Table presenting the proportion of the variance of the dataset explained by main PCs; Table S5: Descriptive statistics of the score distribution of the first four PCs; Table S6: Cross-table analysis comparing the ATTRwt and hATTR groups with the demographic and quantile distribution of four main PC scores; Figure S1: Score distribution of the first four main principal components (PCs); Figure S2: Plot representing the distribution of men and women according to BMI categories.
