# Supplementary material for: Hereditary Transthyretin-Related Amyloidosis Ongoing Observational Study: A Baseline Report of the First 3167 Participants
Source: J Clin Med. 2024 Oct 17;13(20):6197. doi: 10.3390/jcm13206197 (PMC11508262; doi:10.3390/jcm13206197)

SUPPLEMENTARY MATERIAL

Hereditary Transthyretin-related Amyloidosis ongoing clinical study: a baseline report of the first 3,167 participants

Sabine Rösner[1*], Luba M. Pardo[1*], Aida M. Bertoli-Avella[1**], Volha Skrahina[1**], Pierre Engel[1], Sabine Schröder[1], Susan Zielske[1], Valerie Bonke[1], Janett Kreth[1], Gina Westphal[1], Felix Reder[1,] Snezana Skobalj[1], Susanne Zielke[1], Xenia Bogdanovic[1], Paula Grieger[1], Jörg Rennecke[1], Thomas Skripuletz[2,3], Monica Patten[4], Birgit Aßmus[5], Katrin Hahn[6,7] Arndt Rolfs[1,8***,] Peter Bauer[1,9***], TRAM study group

*shared first author

**shared second author

***shared last author

[1]CENTOGENE GmbH, Rostock, Germany

[2]Department of Neurology, Hannover Medical School, Hannover, Germany

[3]Amyloidosis Center of Lower Saxony, Hannover Medical School, Hannover, Germany

[4] Department of Cardiology, University Heart and Vascular Center, Hamburg, Germany

[5] Department of Cardiology and Angiology, University Hospital Giessen and Marburg, Germany

[6] Department of Neurology, Charitè–Universitätsmedizin, Amyloidosis Center Charité Berlin, Berlin, Germany

[7]Amyloidosis Center Charité Berlin (ACCB), Charité Universitätsmedizin Berlin, Germany

[8] RCV GmbH, Institute for Rare Disease Diagnostics, Berlin, Germany

[9] University Rostock, Medical Faculty, Rostock, Germany

TRAM study group principal investigators (in alphabetical order):

Stephan Achenbach, Universitätsklinikum Erlangen, Medizinische Klinik2, Kardiologie und Angiologie, Erlangen; Fabian aus dem Siepen, Heidelberg Universitätsklinikum, Klinik für Kardiologie, Angiologie und Pneumologie, Heidelberg; Tobias Back, Sächsisches Krankenhaus Arnsdorf, Klinik für Neurologie und Neurologische Intensivmedizin, Arnsdorf; Marcus Bauer, St. Vincenz Krankenhaus Datteln, Medizinische Klinik II, Datteln; Petra Baum, Klinische Neurophysiologie, Universitätsklinikum Leipzig - AöR, Klinik und Poliklinik für Neurologie, Leipzig; Jörg Berrouschot, Klinikum Altenburger Land GmbH, Klinik für Neurologie, Altenburg; Achim Berthele, Klinik und Poliklinik für Neurologie, Klinikum rechts der Isar der TU München, München; Frank Birklein, Johannes Gutenberg-Universität Mainz, Klinik und Poliklinik für Neurologie, Mainz; Michael Brandt, UKRB Universitätsklinikum Ruppin Brandenburg, MVZ Kardiologie, Neuruppin; Felix Butscheid, Krankenhaus Buchholz, Fachabteilung Neurologie, Buchholz; Alessandro Cuneo, Klinikum Westmünsterland GmbH, I. Medizinische Klinik, Kardiologie, Angiologie und Diabetologie, Ahaus; Frauke Czepluch, Universitätsmedizin Göttingen – Herzzentrum, Göttingen; Alexander Dressel, Carl-Thiem-Klinikum Cottbus gGmbH, Klinik für Neurologie, Cottbus; Thomas Duning, Gesundheit Nord Klinikverbund Bremen, Klinikum Bremen-Ost, Klinik für Neurologie mit Institut für klinische Neurophysiologie und Neurologische Frührehabilitation, Bremen; Hans Ullrich, Ebersberger, Kardiologie München Nord, München; Philipp Ettelt, Allgemeines Krankenhaus Celle, Neurologische Klinik, Celle; Christian Geber, DRK Schmerz-Zentrum, Mainz; Burkhard Gess, Neurologische Klinik Bethel, Evangelisches Klinikum Bethel gGmbH, Bielefeld; Tobias Graf, Medizinische Klinik II (Kardiologie Angiologie Intensivmedizin), Universitäres Herzzentrum Lübeck, Lübeck; Alexander Grimm, Universitätsklinikum Tübingen, Zentrum für Neurologie, Neurologische Klinik, Poliklinik und Hertie-Institut für klinische Hirnforschung, Tübingen; Albert Grüger, Martin Gropius Krankenhaus, Klinik für Neurologie, Eberswalde; Katrin Hahn, Universitätsmedizin Charité, Klinik für Neurologie/Neurologische Hochschulambulanz, Amyloidosis Center Charité Berlin (ACCB), Berlin; Klaus Hertting, Krankenhaus, Buchholz, Sektion Kardiologie und Angiologie, Buchholz; Fabian Knebel, Universitätsmedizin Charité, Medizinische Klinik mit Schwerpunkt Kardiologie und Angiologie, Berlin; Karl Christian Knop, Neurologie Neuer Wall, Fachärzte für Neurologie & Psychiatrie, Hamburg; Richard Kobza, Luzerner Kantonspital, Kardiologie, Luzern; Markus Krämer, Alfried-Krupp-Krankenhaus, Klinik für Neurologie, Essen; Heidrun Krämer-Best, Neurologische Klinik des Universitätsklinikums Gießen und Marburg GmbH, Standort Gießen, Gießen; Lukas Kremmler, Krankenhaus Barmherzige Brüder Regensburg, Klinik für Neurologie, Regensburg; Albert C. Ludolph, Universitätsklinikum Ulm, Klinik für Neurologie, Ulm; Andrea Maier, Universitätsklinikum Aachen, Klinik für Neurologie, Aachen; Rolf Malessa, Sophien-u. Hufeland-Klinikum, Klinik für Neurologie und Klinische Neurophysiologie, Weimar; Matthias Maschke, Krankenhaus der Barmherzige Brüder Trier, Neurologie und Neurophysiologie, Trier; Sabine Mehnert, Sana HANSE Klinikum Wismar, Klinik für Neurologie, Wismar; Heiko Methe, Kliniken an der Paar Friedberg Krankenhaus – Kardiologie, Friedberg; Tobias Müller, Ruppiner Kliniken GmbH, Hochschulklinikum der MHB, Klinik für Neurologie, Neuruppin; Herbert Naegele, Albertinen-Krankenhaus, Kardiologie, Hamburg; Peter Oberst, Neckar-Odenwald Kliniken gGmbH, Kreiskrankenhaus Mosbach, Kardiologie der Klinik für Innere Medizin, Mosbach; Nikolaos Pagonas, UKRB Universitätsklinikum Ruppin Brandenburg, Medizinische Klinik A, Neuruppin; Monica Patten-Hamel, Universitäres Herzzentrum Hamburg, Klinik für Allgemeine und Interventionelle Kardiologie, Universitätsklinikum Hamburg-Eppendorf, Hamburg; Marko Petrick, Kreiskrankenhaus Prignitz gGmbH, Perleberg; Gernot Reimann, Stroke-Unit und Neurologische Intensivstation, Klinikum Dortmund gGmbH, Dortmund; Andreas Rieth, Kerckhoff-Klinik GmbH, Klinik für Kardiologie, Bad Nauheim; Christian Roth, DRK-Kliniken Nordhessen gGmbH, Klinik für Neurologie und Klinische

Neurophysiologie, Kassel; Volker Schächinger, Herz-Thorax-Zentrum Fulda, Klinikum Fulda gAG, Universitätsmedizin Marburg - Campus Fulda, Fulda; Beate Schlotter-Weigel, Friedrich-Baur-Institut, Neurologische Klinik und Poliklinik, Ludwig-Maximilians-Universität, München; Holger Schmidt, Elbe Klinikum Stade, Klinik für Neurologie, Stade; Ilka Schneider, Klinikum St. Georg gGmbH, Klinik für Neurologie, Leipzig; Florian Schöberl, Neurologische Klinik und Poliklinik & Deutsches Schwindel- und Gleichgewichtszentrum DSGZ, Ludwig-Maximilians-Universität München, München; Herbert Schreiber, NeuroPoint GmbH, Ulm; Paul Christian Schulze, Universitätsklinikum Jena – Kardiologie, Jena; Matthias Schwab Universitätsklinikum Jena, Klinikum für Neurologie, Jena; Bernhard Sehm, Universitätsklinik und Poliklinik für Neurologie, Universitätsklinikum Halle, Halle; Rüdiger Seitz, LVR Klinikum Düsseldorf, Neurologie, Düsseldorf; Thomas Skripuletz, Medizinische Hochschule Hannover, Klinik für Neurologie, Hannover; Hassan Soda, Rhön-Klinikum, Klinik für Neurologie und Neurologische Intensivmedizin, Bad Neustadt; Anne-Dorte Sperfeld, Sächsisches Krankenhaus Altscherbitz, Fachkrankenhaus für Psychiatrie und Neurologie, Schkeuditz; Christian Reiter, Kepler Universitätsklinikum, Klinik für Interne 1 - Kardiologie und internistische Intensivmedizin, Linz; Christoph Stellbrink, Klinikum Bielefeld, Klinik für Kardiologie und internistische Intensivmedizin, Bielefeld; Maria Tafelmeier, Universitätsklinikum Regensburg, Kardiologie, Regensburg; Christian Tanislav, Jung-Stilling Klinikum Siegen, Diakonie Westfalen Süd, Neurogeriatrie, Siegen; Adrian Trommer, Fachkrankenhaus Hubertusburg gGmbH, Neurologische Klinik, Wermsdorf; Carsten Tschöpe, Universitätsmedizin Charité, Klinik für Innere Medizin mit Schwerpunkt Kardiologie Charité, Campus Virchow Klinikum (CVK), Berlin; Jochen C. Ulzheimer, Gemeinschaftspraxis Neurologie und Psychiatrie im Stadtpalais Aschaffenburg, Aschaffenburg; Rolf Vogel, Kantonsspital Olten - Solothurner Spitäler, Kardiologie, Olten; Alexander Vogt, Universitätsklinik und Poliklinik für Innere Medizin III, Halle; Tim von Oertzen, Kepler Universitätsklinikum, Neurologie, Neuromed Campus, Linz; Martin Wambach, Klinik für Kardiologie und Angiologie, Contilia Herz- und Gefäßzentrum, Elisabeth-Krankenhaus Essen, Essen; Joachim Weil, Sana Kliniken Lübeck GmbH, Medizinische Klinik II, Lübeck; Joachim Wolf, Diakonissen Speyer-Mannheim, Diakonissenkrankenhaus Mannheim, Klinik für Neurologie, Mannheim

Table S1. Centers participating in the study

City	Institution	Main clinician involved
**Aachen**	Universitätsklinikum Aachen, Klinik für Neurologie	Dr. Andrea Maier
**Ahaus**	Klinikum Westmünsterland GmbH, I. Medizinische Klinik, Kardiologie, Angiologie und Diabetologie	Dr. Alessandro Cuneo
**Altenburg**	Klinikum Altenburger Land GmbH, Klinik für Neurologie	Prof. Dr. Jörg Berrouschot
**Arnsdorf**	Sächsisches Krankenhaus Arnsdorf, Klinik für Neurologie und Neurologische Intensivmedizin	Prof. Dr. Tobias Back
**Aschaffenburg**	Gemeinschaftspraxis Neurologie und Psychiatrie im Stadtpalais Aschaffenburg	Dr. Jochen C. Ulzheimer
**Bad Nauheim**	Kerckhoff-Klinik GmbH, Klinik für Kardiologie	Dr. Andreas Rieth
**Bad Neustadt**	Rhön-Klinikum, Klinik für Neurologie und Neurologische Intensivmedizin	Dr. Hassan Soda
**Berlin**	Universitätsmedizin Charité, Klinik für Neurologie/Neurologsiche Hochschulambulanz, Amyloidosis Center Charité Berlin (ACCB)	PD Dr. Katrin Hahn
**Berlin**	Universitätsmedizin Charité, Medizinische Klinik mit Schwerpunkt Kardiologie und Angiologie	Prof. Dr. Fabian Knebel
**Berlin**	Universitätsmedizin Charité, Klinik für Innere Medizin mit Schwerpunkt Kardiologie Charité, Campus Virchow Klinikum (CVK)	Prof. Dr. Carsten Tschöpe
**Bielefeld**	Neurologische Klinik Bethel, Evangelisches Klinikum Bethel gGmbH	Dr. Burkhard Gess; Prof. Dr. Wolf-Rüdiger Schäbitz
**Bielefeld**	Klinikum Bielefeld, Klinik für Kardiologie und internistische Intensivmedizin	Prof. Dr. Christoph Stellbrink
**Bremen**	Gesundheit Nord Klinikverbund Bremen, Klinikum Bremen-Ost, Klinik für Neurologie mit Institut für klinische Neurophysiologie und Neurologische Frührehabilitation	Prof. Dr. Thomas Duning
**Buchholz**	Krankenhaus Buchholz, Sektion Kardiologie und Angiologie	Dr. Klaus Hertting
**Buchholz**	Krankenhaus Buchholz, Fachabteilung Neurologie	Dr. Felix Butscheid
**Celle**	Allgemeines Krankenhaus Celle, Neurologische Klinik	Dr. Philipp Ettelt; Prof. Dr. Wolfgang Heide
**Cottbus**	Carl-Thiem-Klinikum Cottbus gGmbH, Klinik für Neurologie	Prof. Dr. Alexander Dressel

**Datteln**	St. Vincenz Krankenhaus Datteln, Medizinische Klinik II	Dr. Marcus Bauer
**Dortmund**	Stroke-Unit und Neurologische Intensivstation, Klinikum Dortmund gGmbH	Dr. Gernot Reimann
**Düsseldorf**	LVR Klinikum Düsseldorf, Neurologie	Prof. Dr. Rüdiger Seitz
**Eberswalde**	Martin Gropius Krankenhaus, Klinik für Neurologie	Dr. Albert Grüger
**Erlangen**	Universitätsklinikum Erlangen, Medizinische Klinik2, Kardiologie und Angiologie	Prof. Dr. Stephan Achenbach
**Essen**	Alfried-Krupp-Krankenhaus, Klinik für Neurologie	PD Prof. Markus Krämer
**Essen**	Klinik für Kardiologie und Angiologie, Contilia Herz- und Gefäßzentrum, Elisabeth-Krankenhaus Essen	Dr. Jan Martin Wambach; Dr. Katharina Hellhammer; Dr. Robert Schueler
**Friedberg**	Kliniken an der Paar Friedberg Krankenhaus – Kardiologie,	PD Dr. Heiko Methe
**Fulda**	Herz-Thorax-Zentrum Fulda, Klinikum Fulda gAG, Universitätsmedizin Marburg - Campus Fulda	Prof. Dr. Volker Schächinger
**Gießen**	Neurologische Klinik des Universitätsklinikums Gießen und Marburg GmbH, Standort Gießen	Prof. Dr. Heidrun Krämer-Best; Dr. Martin Jünemann
**Göttingen**	Universitätsmedizin Göttingen – Herzzentrum	Dr. Frauke Czepluch
**Halle**	Universitätsklinik und Poliklinik für Innere Medizin III	Dr. Alexander Vogt; Dr. Marios Matiakis; Prof. Dr. Michel Noutsias
**Halle**	Universitätsklinik und Poliklinik für Neurologie, Universitätsklinikum Halle	PD Dr. Bernhard Sehm
**Hamburg**	Albertinen-Krankenhaus, Kardiologie	Prof. Dr. Herbert Naegele
**Hamburg**	Neurologie Neuer Wall, Fachärzte für Neurologie & Psychiatrie	Dr. Karl Christian Knop
**Hamburg**	Universitäres Herzzentrum Hamburg, Klinik für Allgemeine und Interventionelle Kardiologie, Universitätsklinikum Hamburg-Eppendorf	Prof. Monica Patten-Hamel
**Hannover**	Medizinische Hochschule Hannover, Klinik für Neurologie	Prof. Dr. Thomas Skripuletz
**Heidelberg**	Heidelberg Universitätsklinikum, Klinik für Kardiologie, Angiologie und Pneumologie	Dr. Fabian aus dem Siepen
**Jena**	Universitätsklinikum Jena, Klinikum für Neurologie	Prof. Dr. Matthias Schwab; Prof. Dr. Otto W. Witte
**Jena**	Universitätsklinikum Jena – Kardiologie	Prof. Dr. Paul Christian Schulze

**Kassel**	DRK-Kliniken Nordhessen gGmbH, Klinik für Neurologie und Klinische Neurophysiologie	PD Dr. Christian Roth
**Leipzig**	Klinische Neurophysiologie, Universitätsklinikum Leipzig - AöR, Klinik und Poliklinik für Neurologie	Prof. Dr. Petra Baum
**Leipzig**	Klinikum St. Georg gGmbH, Klinik für Neurologie	Dr. Ilka Schneider
**Linz**	Kepler Universitätsklinikum, Klinik für Interne 1 - Kardiologie und internistische Intensivmedizin	Dr. Christian Reiter
**Linz**	Kepler Universitätsklinikum, Neurologie, Neuromed Campus	Dr. Tim von Oertzen
**Luzern**	Luzerner Kantonspital, Kardiologie	PD Dr. Richard Kobza
**Lübeck**	Medizinische Klinik II (Kardiologie Angiologie Intensivmedizin), Universitäres Herzzentrum Lübeck	Dr. Tobias Graf
**Lübeck**	Sana Kliniken Lübeck GmbH, Medizinische Klinik II	Prof. Dr. Joachim Weil
**Mainz**	DRK Schmerz-Zentrum	Dr. Christian Geber
**Mainz**	Johannes Gutenberg-Universität Mainz, Klinik und Poliklinik für Neurologie	Prof. Dr. Frank Birklein
**Mannheim**	Diakonissen Speyer-Mannheim, Diakonissenkrankenhaus Mannheim, Klinik für Neurologie	PD Dr. Joachim Wolf
**Mosbach**	Neckar-Odenwald Kliniken gGmbH, Kreiskrankenhaus Mosbach, Kardiologie der Klinik für Innere Medizin	Dr. Peter A. Oberst
**München**	Klinik und Poliklinik für Neurologie, Klinikum rechts der Isar der TU München	Prof. Dr. Achim Berthele
**München**	Kardiologie München Nord	Dr. Hans Ullrich Ebersberger
**München**	Friedrich-Baur-Institut, Neurologische Klinik und Poliklinik, Ludwig-Maximilians-Universität	Dr. Beate Schlotter-Weigel
**München**	Neurologische Klinik und Poliklinik & Deutsches Schwindel- und Gleichgewichtszentrum DSGZ, Ludwig-Maximilians-Universität München	Dr. Florian Schöberl; Dr. Ozan Emre Eren; Prof. Dr. Andreas Straube
**Neuruppin**	Ruppiner Kliniken GmbH, Hochschulklinikum der MHB, Klinik für Neurologie	Dr. Tobias Müller
**Neuruppin**	UKRB Universitätsklinikum Ruppin Brandenburg, MVZ Kardiologie	Michael Brandt
**Neuruppin**	UKRB Universitätsklinikum Ruppin Brandenburg, Medizinische Klinik A	Prof. Dr. Nikolaos Pagonas
**Olten**	Kantonsspital Olten - Solothurner Spitäler, Kardiologie	Prof. Dr. Rolf Vogel
**Perleberg**	Kreiskrankenhaus Prignitz gGmbH	Dr. Marko Petrick
**Regensburg**	Krankenhaus Barmherzige Brüder Regensburg, Klinik für Neurologie	Dr. Lukas Kremmler

**Regensburg**	Universitätsklinikum Regensburg, Kardiologie	PD Dr. Maria Tafelmeier; Prof. Dr. Bernhard Unsöld
**Schkeuditz**	Sächsisches Krankenhaus Altscherbitz, Fachkrankenhaus für Psychiatrie und Neurologie	PD Dr. Anne-Dorte Sperfeld
**Siegen**	Jung-Stilling Klinikum Siegen, Diakonie Westfalen Süd, Neurogeriatrie	Prof. Dr. Christian Tanislav
**Stade**	Elbe Klinikum Stade, Klinik für Neurologie	Prof. Dr. Holger Schmidt
**Trier**	Krankenhaus der Barmherzige Brüder Trier, Neurologie und Neurophysiologie	Prof. Dr. Matthias Maschke
**Tübingen**	Universitätsklinikum Tübingen, Zentrum für Neurologie, Neurologische Klinik, Poliklinik und Hertie-Institut für klinische Hirnforschung	Prof. Alexander Grimm
**Ulm**	NeuroPoint GmbH	Prof. Dr. Herbert Schreiber
**Ulm**	Universitätsklinikum Ulm, Klinik für Neurologie	Prof. Dr. Albert C. Ludolph
**Weimar**	Sophien-u. Hufeland-Klinikum, Klinik für Neurologie und Klinische Neurophysiologie	PD Dr. Rolf Malessa
**Wermsdorf**	Fachkrankenhaus Hubertusburg gGmbH, Neurologische Klinik	Dr. Adrian Trommer
**Wismar**	Sana HANSE Klinikum Wismar, Klinik für Neurologie	Dr. Sabine Mehnert

Table S2. Descriptive statistics of main symptoms used for PCA.

Descriptive Statistics			
**Clinical symptoms**	Mean	Std. Deviation	Analysis N*
**Dizziness**	1.60	.543	3112
**Difficulty to hold an erection**	2.18	.668	3112
**Diarrhoea**	1.88	.407	3112
**Constipation**	1.84	.445	3112
**Bouts of constipation/diarrhoea**	1.94	.360	3112
**Difficulty to hold urine**	1.84	.529	3112
**Unintended weight loss**	1.86	.408	3112
**Heartbeat alterations**	1.74	.522	3112
**Sense temperature dysregulation**	1.75	.558	3112
**Burning feet**	1.64	.532	3112
**Carpal tunnel symptoms**	1.76	.538	3112
**Dyshidrosis**	1.90	.496	3112
**Anaemia**	1.96	.394	3112
**Allodynia**	1.91	.486	3112
**Shortness of breath**	1.60	.545	3112
**Palpitations**	1.75	.506	3112
**Water retention in limbs**	1.67	.534	3112
**Chest pain**	1.86	.409	3112
**Numbness/Tingling**	1.39	.513	3112

*Based on complete cases

Table S3. Test that assesses whether the data are suitable for PCA.

KMO and Bartlett's Test		
Kaiser-Meyer-Olkin Measure of Sampling Adequacy.		.807
Bartlett's Test of Sphericity	Approx. Chi-Square	9129.994
	df	171
	Sig.	.000

Table S4. Table presenting the proportion of the variance of the dataset explained by main PCs. PCs with an eigen value>1 were retained as relevant components.

**Total Variance Explained**									
**Component**	Initial Eigenvalues			Extraction Sums of Squared Loadings			Rotation Sums of Squared Loadings		
	Total	% of Variance	Cumulative %	Total	% of Variance	Cumulative %	Total	% of Variance	Cumulative %
**1**	3.474	18.285	18.285	3.474	18.285	18.285	2.340	12.316	12.316
**2**	2.276	11.977	30.262	2.276	11.977	30.262	2.110	11.104	23.420
**3**	1.366	7.191	37.453	1.366	7.191	37.453	1.806	9.505	32.925
**4**	1.145	6.029	43.482	1.145	6.029	43.482	1.777	9.353	42.278
**5**	1.031	5.425	48.907	1.031	5.425	48.907	1.260	6.629	48.907
**6**	.965	5.078	53.985						
**7**	.885	4.657	58.642						
**8**	.856	4.507	63.150						
**9**	.811	4.271	67.420						
**10**	.811	4.268	71.688						
**11**	.775	4.079	75.768						
**12**	.704	3.705	79.473						
**13**	.641	3.372	82.845						
**14**	.618	3.253	86.098						
**15**	.595	3.133	89.230						
**16**	.584	3.074	92.305						
**17**	.542	2.850	95.155						
**18**	.480	2.526	97.681						
**19**	.441	2.319	100.000						
**Extraction Method: Principal Component Analysis.**									

Table S5. Descriptive statistics of the score distribution of 4 first PCs. Quantiles were used to categorize the PCs into 4 groups to facilitate comparisons.

	PC-1(PN)	PC-2(CM)	PC-3(GI)	PC-4(GS)	PC-5(CT)
Total	2345	2345	2345	2345	2345
Missing	861	861	861	861	861
Mean	0	0	0	0	0
Minimum	-2.619	-2.921	-3.961	-4.039	-2.943
Maximum	1.689	1.818	1.233	1.701	2.349
Percentile					
25	-0.790	-0.691	-0.229	-0.067	-0.696
50	0.241	0.294	0.383	0.363	0.168
75	0.893	0.796	0.578	0.615	0.793

Table S6. *Cross table analysis comparing quantile distribution of four main PC scores with different demographics and clinical variables.* *Observed and expected (in brackets) number of participants per cell are presented. Chi-squared test (X^2) tests overall differences in the distribution of counts of quantile (Q) per variable. Significant differences in the distribution per cell adjusted for multiple testing are highlighted in bold. Significant differences were observed per gender for PC-PN; PC-CM and PC-GI.*

	**PC-PN**				**X^2; 3 df (P-value)**	**PC-CM**				**X^2; 3 df; (P-value)**	**PC-GI**				**X^2; 3 df; (P-value)**	**PC-GC**				**X^2; 3 df (P-value)**
**Gender**	Q1	Q2	Q3	Q4	55.7; (4.9e^{-12})	Q1	Q2	Q3	Q4	18.13; (4.0e-3)	Q1	Q2	Q3	Q4	55.8; (4.7e-12)	Q1	Q2	Q3	Q4	9.2; (0.03)
**Men**	**329 (385)**	**354 (381)**	**413 (381)**	**433 (383)**		**345 (382)**	**414 (384)**	420 (409)	350 (355)		**315 (385)**	**434 (404)**	**394 (355)**	383 (383)		373 (383)	397 (383)	400 (3815)	361 (381)	
**Women**	**260 (204)**	**228 (202)**	**169 (202)**	**153 (203)**		**239 (202)**	**173 (203)**	206 (217)	193 (188)		**273 (203)**	**183 (213.)**	**148 (188)**	202 (202)		213 (213)	189 (189)	182 (182)	227 (227)	
**Fam. PNP**					28.0; (3.6e^{-06})					8.60; (0.04)					12.0; (0.007)					4.86; (0.18)

**Yes**	**86 (55)**	63 (55)	40 (55)	**30 (55)**		47 (55)	42 (55)	72 (59)	58 (51)		**72 (55)**	57 (58)	41 (51)	48 (55)		47 (55)	49 (55)	66 (54)	57 (55)	
**No**	404 (390)	372 (386)	386 (396)	390 (389)		387 (389)	396 (389)	393 (416)	378 (360)		**353 (390)**	413 (410)	372 (360)	411 (388)		382 (388)	401 (390)	369 (386)	402 (391)	
**Fam. CM**					18.8; (0.0003)					35.5; (9.6e-08)					23.4; (3.3e-05)					1.00; (0.80)
**Yes**	**33 (55)**	60 (55)	55 (55)	71 (59)		**87 (55)**	61 (55)	46 (59)	**27 (51)**		**80 (56)**	55 (55)	**32 (59)**	54 (51)		54 (55)	54 (55)	51 (55)	62 (56)	
**No**	**409 (364)**	340 (360)	346 (360)	351 (262)		338 (362)	361 (363)	392 (387)	**356 (335)**		**324 (363)**	388 (382)	**350 (335.)**	379 (361)		360 (361.6)	366 (362.2)	359 (359)	362 (364)	
**Genetic**																				
**TTR**					6.07; (0.11)					6.63; (0.08)					9.3 (0.026)					1.92; (0.59)
**Positive**	8 (15)	16 (15)	14 (15)	21 (15)		7 (15)	15 (15)	18 (16)	19 (14)		20 (15)	21 (16)	12 (14)	6 (15)		16 (15)	18 (15)	14 (15)	11 (15)	
**Negative**	580 (573)	566 (567)	569 (568)	565 (571)		579 (571)	569 (569)	609 (611)	524 (529)		568 (573)	597 (602)	530 (528)	578 (567)		570 (571)	566 (570)	568 (567)	579 (575)	

Expected numbers in brackets rounded to integers

Table S7. Cross table analysis comparing ATTRwt (n=503) and hATTR (n=92) groups with demographic and quantile distribution of four main PC scores**.** Observed values are compared to the expected values (between parenthesis). Values depicted in bold indicate significant differences.

	**ATTRwt**	**hATTR**	**Chi-square test (3df); P-value**
**Gender**			**32.36; 1.28x10^{-8}**
Men	**359 (338)**	**49 (69)**	
Women	76 (96.9)	41 (20.1)	
**PN**			**13.02; p-value = 0.005**
Q1	**13 (18.4)**	**9 (3.6)**	
Q2	67 (69.2)	16 (13.8)	
Q3	116 (108.5)	14 (21.5)	
Q4	111 (111)	22 (22)	
**CM**			**1.59; p-value = 0.009**
Q1	**82 (74.2)**	**7 (14.8)**	
Q2	102 (99.2)	17 (19.8)	
Q3	70 (73.4)	18 (14.6)	
Q4	52 (59.2)	19 (11.8)	
**GI**			6.1; p-value = 0.105
Q1	82 (82)	20 (17)	
Q2	84 (88.4)	22 (17.6)	
Q3	63 (62.5)	12 (12.5)	
Q4	77 (70)	7 (14)	
**GC**			
Q1	106 (101.8)	16 (20.2)	3.63; p-value=0.304
Q2	69 (74.2)	20 (14.8)	
Q3	66 (66.7)	14 (13.3)	
Q4	66 (64.2)	11 (12.8)	
**Fam. PNP***			**81.60; p-value < 2.2x10^{-16}**
Yes	**6 (27.1)**	**26 (4.9)**	
No	271 (263.8)	41(48.2)	
**Fam. CM***			**64.86; p-value = 8.02x10^{-16}**
Yes	**27 (51.6)**	**34 (9.4)**	
No	254 (240)	30 (43.9)	

*For this variable Chi-square was calculated with 1df

SUPPLEMENTARY FIGURES

Supplementary Figure 1. Score distribution of the first four main principal components (PCs) derived from the clinical symptoms and table of number of participants per PC-quantiles. The quantiles were derived from the 1st to 25th- percentile (first quantile); 25th to 50th percentile (second quantile-median), 50th -75th percentile (3rd quantile) and 75th-100th percentile (4th quantile). Thresholds were presented in Supplementary Table 5.

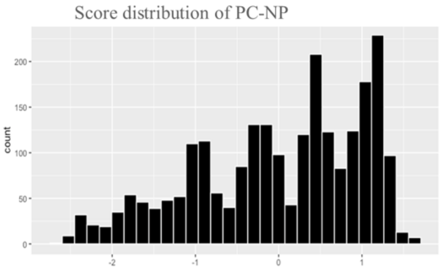

Quantile distribution PC-NP	*Number of people per quantile*	*Percent*
First quantile	589	18.4
Second quantile	583	18.2
Third quantile	583	18.2
Fourth Quantile	587	18.3
Missing	864	26.9
**Total**	**3206**	**100.0**

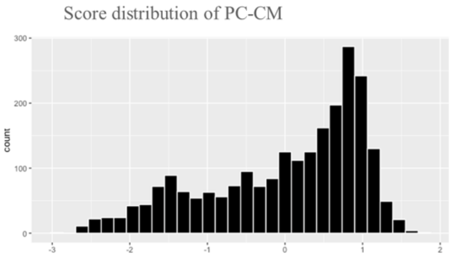

Quantile distribution PC-CM	*Number of people per quantile*	*Percent*
First quantile	**586**	**18.3**
Second quantile	**587**	**18.3**
Third quantile	**627**	**19.6**
Fourth Quantile	**543**	**16.9**
Missing	**2343**	**73.1**
Total	**3206**	**100.0**

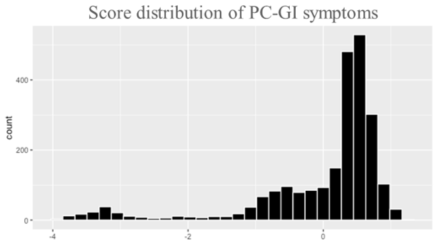

Quantile distribution PC-GI	*Number of people per quantile*	*Percent*
First quantile	**588**	**18.3**
Second quantile	**619**	**19.3**
Third quantile	**543**	**16.9**
Fourth Quantile	**585**	**18.2**
Missing	**871**	**27.2**
**Total**	**3206**	**100.0**

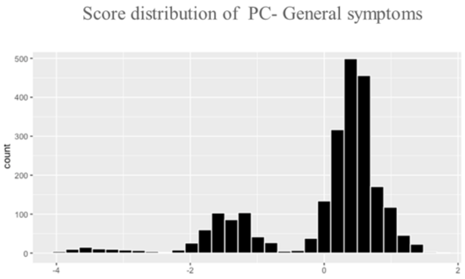

Quantile distribution PC-GS	*Number of people per quantile*	*Quantile distribution PC-GI*
First quantile	**586**	**18.3**
Second quantile	**587**	**18.3**
Third quantile	**582**	**18.2**
Fourth Quantile	**590**	**18.4**
Missing	**861**	**26.9**
**Total**	**3206**	**100.0**

Supplementary Figure 2. Plot representing the distribution of men (1) and women (2) per BMI categories. Category thresholds of weight are defined according to standard criteria for body mass index (BMI).

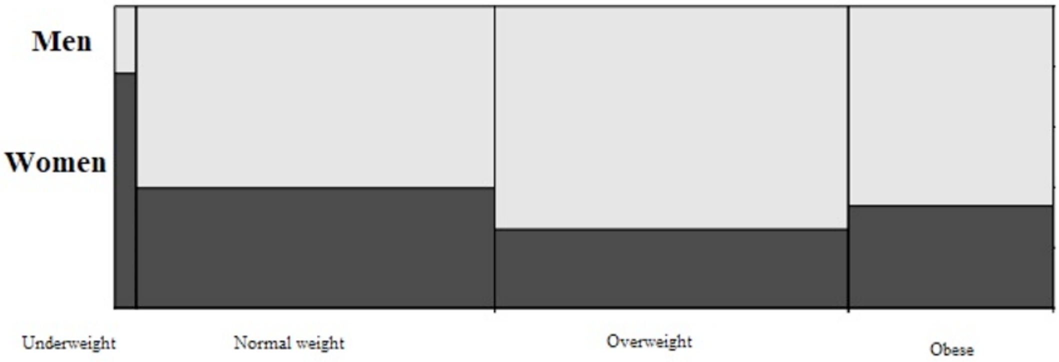

Supplement: Supplementary file 1 [file jcm-13-06197-s001.zip › jcm-3229752-supplementary.pdf]
